# Aurodox inhibits type III secretion in multiple Gram-negative pathogens

**DOI:** 10.1098/rsob.240240

**Published:** 2024-11-27

**Authors:** David R. Mark, Nicky O’Boyle, Kabo R. Wale, Samantha K. Tucker, Rebecca E. McHugh, Andrew J. Roe

**Affiliations:** ^1^ School of Infection and Immunity, University of Glasgow, Glasgow G12 8TA, UK; ^2^ Department of Pathology, School of Microbiology & School of Medicine, University College Cork, National University of Ireland, Cork T12 K8AF, Ireland; ^3^ Department of Microbiology, School of Genetics & Microbiology, Moyne Institute of Preventive Medicine, Trinity College Dublin, Dublin D02 A2H0, Ireland; ^4^ Biological Sciences, University of Botswana, Gaborone, Botswana

**Keywords:** aurodox, polyketide, virulence, type 3 secretion system, infection

## Abstract

Gram-negative pathogens pose a significant threat due to their propensity for causing various infections, often coupled with formidable resistance to conventional antibiotic treatments. The development of antivirulence (AV) compounds emerges as a promising alternative strategy by disrupting virulence mechanisms rather than targeting bacterial viability. Aurodox has exhibited promising AV properties in previous studies by blocking the expression and function of the LEE-encoded type 3 secretion system (T3SS) in enterohaemorrhagic *Escherichia coli*, an injectosome that translocates effector proteins directly into host target cells. However, aurodox’s efficacy against the T3SS of other pathogens remained unanswered. Using quantitative real-time polymerase chain reaction, we show that aurodox exerts inhibitory effects on selected T3SS including those of *Salmonella* Typhimurium, *Yersinia pseudotuberculosis* and *Vibrio parahaemolyticus*. Imaging of RAW 264.7 cells infected with *S*. Typhimurium showed that aurodox protects against late stages of infection by blocking the expression of the SPI-2 T3SS. To elucidate a conserved mechanism of action, we compared transcriptomic datasets from both *E. coli* and *S*. Typhimurium treated with aurodox to identify orthologous genes differentially expressed in response to aurodox treatment across both pathogens. This study sheds light on potential mechanisms driving the action of this promising AV compound.

## 1. Introduction

Gram-negative pathogens (GNPs) pose a serious threat to public health due to their ability to cause a range of infections while exhibiting high levels of resistance to antibiotic therapies [1]. One potential strategy to treat GNPs is the use of antivirulence (AV) compounds. The mode of action of these compounds differs from traditional antibiotics as they inactivate virulence mechanisms instead of growth inhibition or killing. One such AV target is the type 3 secretion system (T3SS), a needle-like injectosome structure that is deployed to translocate effector proteins from the bacteria to the host target cells [2,3].

## 2. Material and methods

### 2.1. Acquisition and storage of aurodox

Aurodox was purchased in its pure form (BOC Sciences, Shirley, NJ, USA). A 1 mg ml^−1^ stock solution was prepared by dissolving the compound in dimethyl sulfoxide (DMSO). The overall concentration of DMSO was <0.5% in all experiments.

### 2.2. Growth of bacterial strains in T3SS-inducing conditions for quantitative reverse transcription polymerase chain reaction

Overnight Lysogeny Broth (LB) cultures were diluted one-hundred-fold in T3SS-inducing media (electronic supplementary material, table S1). Cultures were incubated at 37°C, 200 r.p.m. for 6 h before measuring the OD600 nm and collecting approximately 10^9^ cells by centrifugation at 16 000 *× g* for 2 min.

### 2.3. mRNA extraction

Following growth in T3SS-inducing conditions (electronic supplementary material, table S3), cells were harvested by centrifugation, resuspended in two volumes of RNA protect reagent (Qiagen, CA, USA) and incubated for 10 min at room temperature. Following incubation, cells were pelleted by centrifugation at 16 000 *× g* for 10 min and the supernatant was discarded. Total RNA was extracted by using a PureLink RNA Mini Kit (Thermo Fisher Scientific) according to the manufacturer’s instructions after which TurboDNase treatment (Thermo Fisher Scientific) was used to remove contaminating DNA from the samples. For the concentration of extracted RNA, the phenol : chloroform method was used. Briefly, RNA samples were brought to a total volume of 400 μl with nuclease-free water (NucH_2_O) and then mixed with an equal volume of phenol : chloroform : isoamyline alcohol 2 : 24 : 1 (PCIA, Sigma Aldrich). The samples were then centrifuged at room temperature at 13 000 × *g* for 5 min, then the upper layer solution was removed and placed in a clean 1.5 ml Eppendorf tube. Then 400 μl of chloroform : isolamyl alcohol 24 : 1 (CIA, Sigma Aldrich) was added to the upper layer in a clean 1.5 ml Eppendorf tube, and the tube was vortexed briefly and centrifuged for an additional 1 min at maximum speed. After centrifugation, the upper layer was removed and transferred to a clean Eppendorf tube containing 1 μl GlycoBlue coprecipitate (Thermo Fisher Scientific), 40 μl sodium acetate and 800 μl 100% ethanol. Samples were vortexed and stored at −80°C. Following incubation samples were centrifuged at 13 000 × *g* for 20 min at 4°C. The supernatant was removed, and the samples were centrifuged for 5 min at 13 000 *× g* after adding 1 ml of 70% ethanol. The supernatant was removed, and the pellet was air dried. Finally, the pellet was resuspended in 50 μl of NucH_2_O, and the concentration was measured using a NanoDrop DS-11 spectrophotometer (DeNovix).

### 2.4. Quantitative reverse transcription polymerase chain reaction analysis

Ten ng of DNA-free mRNA was used to synthesize 10 μl cDNA with the LunaScript RT Supermix kit (NEB). One microlitre of cDNA was used as a template in quantitative reverse transcription polymerase chain reaction (qRT-PCR) reactions using Luna Universal qPCR Master mix (NEB). The following conditions were employed on a Biorad CFX96 thermocycler: initial denaturation at 95°C for 3 min, followed by 39 cycles of 15 s denaturation and 30 s extension at 60°C. Expression was calculated as 2^-ΔΔct^ relative to expression in DMSO, with *groEL* selected as a housekeeping gene control.

### 2.5. T3SS phylogeny

We hypothesized that evolutionary conservation may play a role in our observation that not all tested T3SS were inhibited by aurodox, and so we sought to test this by placing them in their phylogenetic context. To do this, we downloaded the protein FASTA data for the strains we tested from NCBI, as well as those of organisms that encode T3SS encompassing previously identified phylogroups (as identified from the Secreton database: http://secreton.web.pasteur.fr). These were searched for T3SS using TXSScan as implemented in the Institut Pasteur Galaxy server (https://galaxy.pasteur.fr/). After annotation, the results of TXSScan were manually curated to correct genes that had been assigned to incorrect systems. The amino acid sequences of SctC, SctJ, SctN, SctQ, SctR, SctS, SctT, SctU and SctV from the remaining T3SS were aligned using the ClustalW algorithm as implemented in the msa package in R (v. 1.32.0). These alignments were then concatenated, using tools provided by the Biostrings (v. 2.68.1) and tidyverse (v. 2.0.0) packages. Finally, a maximum-likelihood tree was constructed using IQ-TREE (Galaxy v. 2.1.2+galaxy2) with ModelFinder and UF Bootstrap (1000 bootstraps). The resultant tree was visualized using the treedataverse package and annotated based on T3SS phylogroup and susceptibility to aurodox.

### 2.6. *In vitro* GFP-fusion reporter assays

To expand upon our observations that aurodox inhibits SPI-2 but not SPI-1 in *Salmonella* Typhimurium, we employed previously characterized GFP-based transcriptional reporters (electronic supplementary material, table S3). To transform *S*. Typhimurium SL1344 with pZEP07, pZEP09, pZEP10 and pAJR70, overnight cultures were diluted 1 : 100 in fresh LB and grown to an OD (600 nm) of 0.4–0.6. Cultures were then washed thrice in ice-cold 10% glycerol and resuspended in a final volume of 50 μl of 10% glycerol. The cells were mixed with 50–100 ng of plasmid, electroporated at 2.5 kV, and recovered in SOC medium for 1 h at 37 °C before plating on LB + 25 μg ml^−1^ chloramphenicol (Cm).

For the reporter assays, overnight cultures were diluted 1/100 in 200 μl of LB broth + Cm, and grown in black, clear-bottom plates (Greiner) for 4 h (37°C, 200 r.p.m.), with OD (600 nm) and GFP (excitation 485 nm, emission 520 nm) measurements taken every 10 min. After this, the cultures were centrifuged at 3273 *× g* for 10 min and resuspended in either SPI-1- or SPI-2-inducing media; +Cm, ±5 μg ml^−1^ aurodox and incubated as above. Gene expression was calculated as fluorescence intensity divided by OD (600 nm), minus the values from pZEP07.

### 2.7. RNA-sequencing and associated analysis

Overnight cultures of *S*. Typhimurium were used to inoculate 10 ml cultures in SPI-2-inducing media (electronic supplementary material, table S3) and grown for 1 h. RNA quality was assessed by using an Agilent Bioanalyzer 2100 with 100 ng μl^−1^ concentration accepted as a threshold for individual samples. Library preparation and sequencing were carried out at the University of Glasgow Polyomics facility. Sequencing was carried out on the Illumina NextSeq 500 platform with at least 10 million 100 bp single-end reads being obtained.

Quality checking of sequencing reads was performed using FastQC (Galaxy v. 0.73+galaxy0). Following this reads were aligned to the *S*. Typhimurium genome (Accession GCA_000210855.2) using BWA-MEM2 (Galaxy v. 2.2.1+galaxy0). Featurecounts (Galaxy v. 2.0.1+galaxy2) was used to assign counts to genes, and subsequently differential expression was calculated using edgeR3 (Galaxy v. 3.36.0+galaxy0), adopting the same cutoff for differential expression as previously [4]. Analysis after this point was carried out in R v. 4.3.1. To calculate read mapping to SPI2, the getReadCountsFromBAM function in cn.mops5 was used (window length = 20), the read.gff function in ape6 was used to import annotation data, and further analysis and visualization were conducted using the tidyverse package.

### 2.8. Identification of orthologous differentially expressed genes in EHEC and *Salmonella*

Reads were reanalysed using the same method as described for the *Salmonella* experiment and subsequently extracted the amino acid sequences of both upregulated and downregulated genes from both datasets. Orthologous proteins in these sets were identified using BLAST RBH (Galaxy v. 0.3.0).

### 2.9. Infection of RAW 264.7 macrophage with *S*. Typhimurium SL1344

For the infections, early passage RAW 264.7 cells were seeded onto 13 mm diameter round coverslips, at a density of 5 × 10^4^ cells ml^−1^ in a 24-well plate (Corning), and incubated for 24 h at 37 °C. *Salmonella* Typhimurium::p*rpsM-gfp* were grown overnight in LB broth + Cm, diluted 1/10 into the same media and incubated at 37°C without shaking until an OD 600 nm of 0.6–0.8. To infect, the *Salmonella* were washed twice in PBS and diluted into RPMI 1640 + Cm ± 5 μg ml^−1^ aurodox or DMSO so as to achieve a concentration of 5 × 10^6^ CFU ml^−1^, converted using 1 OD (600 nm) = 7.5 × 10^8^ CFU ml^−1^. Cells were washed twice using PBS, covered in 1 ml of either treated or untreated media, and centrifuged at 400 r.p.m. for 10 min to associate the bacteria and cells. After 1 h, the plate was washed twice with PBS and replaced with clean media containing chloramphenicol, aurodox or DMSO, and gentamicin (30 μg ml^−1^).

After 1 h, 2 h and 24 h, the infections were terminated and stained for imaging. Cells were washed twice in sterile PBS, then fixed in 4% paraformaldehyde for 20 min at room temperature. After fixation, cells were washed once with PBS and permeabilized with 0.1% Triton X-100 for 5 min, washed and stained with AlexaFluor555-phalloidin (1 : 500 dilution, 1 h shaking in the dark). After staining, coverslips were mounted in VectaShield mounting medium plus DAPI Infections were visualized by fluorescent widefield microscopy, at 400× magnification using a Zeiss Axioimager M1 with three infections performed per condition. Images were scored by the proportion of cells in view that were infected and compared using the Wilcoxon rank-sum test.

## 3. Results

Previously, we reported the antivirulence activity of aurodox, a metabolite produced by *Streptomyces goldiniensis* [4–7]. We showed that aurodox abolishes T3SS activity in EHEC, EPEC and *Citrobacter rodentium*. As the activity of aurodox in these pathogens is dependent on the inhibition of the master virulence regulator, *ler* [6], we had proposed that only pathogens carrying a LEE-encoded, *ler*-regulated T3SS would display susceptibility to the antivirulence effects of aurodox.

We tested the capacity of aurodox to treat additional GNPs that carry phylogenetically distinct T3SSs. These were *Salmonella enterica* ssp. *enterica* serovar Typhimurium (which encodes two distinct T3SSs: SPI-1 and SPI-2), *Yersinia pseudotuberculosis* (which encodes a Ysc-type T3SS) and *Vibrio parahaemolyticus* (which carries two T3SSs: VPTTSS1 and VPTTSS2) [2].

**Figure 1.**
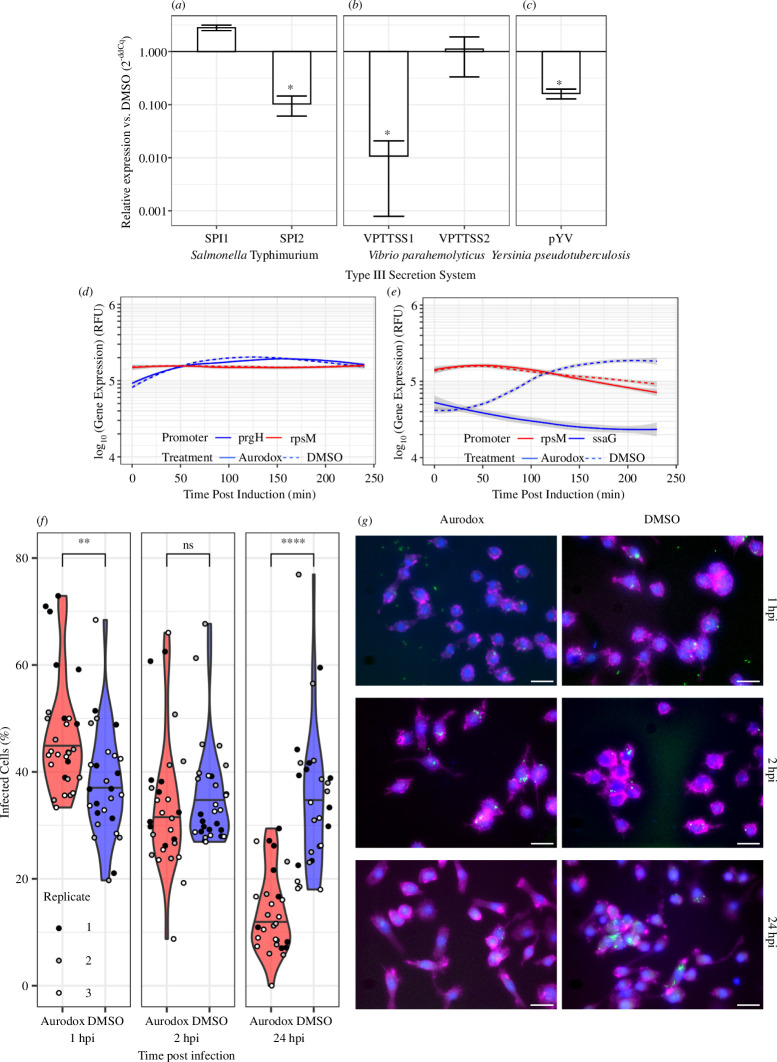
Aurodox inhibits Type III secretion in multiple GNPs. (*a–c*) Quantitative reverse transcription polymerase chain reaction data show that aurodox inhibited the expression of multiple T3SS effectors, including *sseB* of the *Salmonella* Typhimurium SPI-2 T3SS, in SPI-2-inducing conditions (*a*), *vopD* secreted by the *Vibrio parahemolyticus* TTSS1 (*b*) and the pYV-encoded *yopD* of *Yersinia pseudotuberculosis* (*c*). It did not, however, repress expression of *vopD2* of the *V. parahemolyticus* TTSS2 or *sipC* of *Salmonella* Typhimurium’s SPI-1; *p < 0.05 by one-way ANOVA and Tukey’s post hoc test. (*d,e*) Transcriptional reporter assays in *Salmonella* Typhimurium measuring GFP accumulation from the SPI-1-encoded prgH promoter (*d*) or SPI-2-encoded ssaG promoter (*e*). Bacteria were grown to early stationary phase in LB before being switched to inducing media ± aurodox. Aurodox prevented induction of ssaG in SPI-2-inducing media, but had no effect on the expression of *prgH*. (*f*) Violin plots of RAW 264.7 infection efficiency by *Salmonella* Typhimurium::pAJR145 in the presence and absence of aurodox. Horizontal black lines represent the median of each group. One hour post infection, significantly more cells were infected in the aurodox-treated group (*µ* = 48.6% aurodox treated versus 37.8% DMSO treated), there was no difference 2 h post infection (*µ* = 34% aurodox treated versus 35.6% DMSO treated), and significantly fewer cells were infected at 24 h (*µ* = 13.6% aurodox treated versus 34.8%. DMSO treated). **p < 0.01 and ****p < 0.001 by one-way ANOVA and Tukey’s post hoc test; NS, not significant. (*g*) Representative images of treatment groups at different time points. Scale bar, 25 μm.

Expression of specific T3SS effectors in each pathogen in response to aurodox was measured using qRT-PCR (figure 1*a–c*). In *S*. Typhimurium, we observed downregulation of the SPI-2 effector protein *sseB* (figure 1*a*; >23-fold reduction in SPI-2-inducing media, *p* < 0.0001), and marginal upregulation of the expression of the SPI-1 effector *sipC* (0.35-fold change in SPI-1-inducing media, *p* = 0.08). In *Vibrio parahaemolyticus,* aurodox treatment results in a significant reduction in the expression of *vopD* (figure 1*b*; >90-fold reduction, *p* < 0.01) but not *vopD2*. (0.91-fold change, *p* = 0.97). Finally, in *Yersinia pseudotuberculosis,* aurodox downregulated the expression of the Ysc-type effector *yopD* (figure 1*c*; >6-fold reduction, *p* = 0.015). The activity of aurodox spanned multiple pathogens, revealing that it is not limited to LEE-type T3SSs.

The differential repression of SPI-1 and SPI-2 in *S*. Typhimurium was further analysed using transcriptional GFP reporter assays [8]. For SPI-1, expression of the *prgH* promoter was measured over a 4 h time course (figure 1*d*). Similarly, a reporter driven by the *ssaG* promoter was used to measure the transcriptional response of the SPI-2 structural gene to aurodox treatment. Growth of *S*. Typhimurium in SPI-1-inducing media resulted in a three- to four-fold induction of *prgH*, which was unaffected by the addition of aurodox. In comparison, growth in SPI-2 conditions induced *ssaG* 10-fold (DMSO alone), whereas there was complete prevention of induction when aurodox was added (figure 1*e*). The ribosomal promoter, *rpsM*, was used as a control, showing <5% variation between aurodox-treated and untreated cells. The growth of *Salmonella* was not significantly affected until aurodox ≥8 μg ml^−1^, higher than required to inhibit SPI-2 (electronic supplementary material, figure S1). These data confirm the specific inhibitory effect of aurodox on the SPI-2 T3SS in *S*. Typhimurium. During *S*. Typhimurium infection, the SPI-2 T3SS is responsible for maintaining *Salmonella* within *Salmonella*-containing vacuoles (SCV) within intestinal epithelial cells and macrophages. To determine whether aurodox-mediated inhibition of SPI-2 translated to impaired virulence, RAW 264.7 macrophages were infected with *S*. Typhimurium expressing GFP for visualization (figure 1*f,g*). Cells were imaged at 1 h post infection (hpi), 2 hpi and 24 hpi. At 1 hpi, more cells were infected following aurodox treatment than the control (*μ* = 48.6% of cells infected with aurodox treatment versus 37.8% of control cells, *p *< 0.05). At 2 hpi, there was no significant difference (*μ* = 34% versus 35.6%). Conversely, at 24 hpi, fewer cells were infected by *Salmonella* following aurodox treatment (*μ* = 13.6% with aurodox treatment versus 34.8% control, *p *< 0.001). This is consistent with the inhibition of SPI-2-dependent SCV stabilization and demonstrates that aurodox is protective during the intracellular phase of *Salmonella* pathogenesis. The increased infection levels at the earliest timepoint may reflect the upregulation of SPI-1.

**Figure 2.**
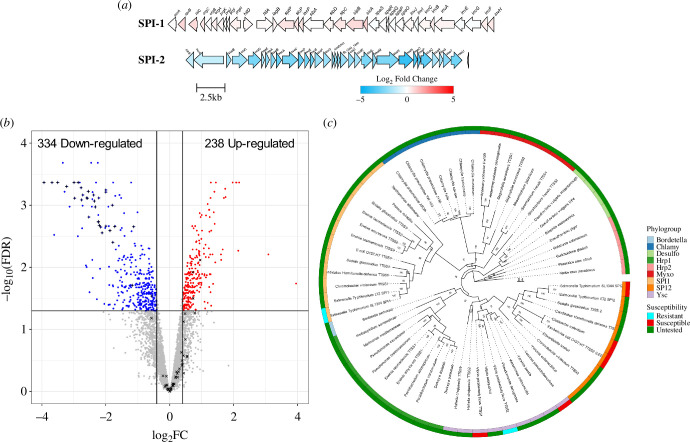
The T3SS inhibiting effects of aurodox may be phylogenetically constrained. (*a*) Volcano plot showing the global effect of aurodox on the *S*. Typhimurium transcriptome. Genes denoted by a plus (+) are encoded on the SPI-2 pathogenicity island, while genes encoded by a cross (x) are encoded on SPI-1. (*b*) Maps of SPI-1 and SPI-2 illustrating the SPI-2-selectivity in aurodox-mediated inhibition of type III secretion. (*c*) Maximum-likelihood phylogenetic tree of selected type III secretion systems, including those tested against aurodox. We observe that all susceptible T3SS were placed into a single clade containing the Ysc and SPI-2 phylogroups. Tree generated using IQ-tree, with ModelFinder and 1000 ultrafast bootstraps.

The differential effect on SPI-2 over SPI-1 raised the question of how aurodox affects transcription more widely across the genome. To investigate this, RNA-Seq of aurodox-treated *S*. Typhimurium was carried out. Cultures were grown in SPI-2-inducing media with either 5 µg ml^−1^ aurodox or DMSO. RNA was extracted from each after 1 h and converted to cDNA for sequencing. Transcripts were mapped to the reference genome and mean fold change and *p*-values were calculated. In response to aurodox treatment, 334 genes were downregulated and 238 upregulated (11.5% of the genome) when compared to the DMSO-treated control (figure 2*a*). Differentially expressed genes were identified within the chromosome and three plasmids (electronic supplementary material, figure S2), revealing all 32 genes encoded within the SPI-2 pathogenicity island were significantly downregulated in response to aurodox (figure 2*b*). These analyses confirm the results of qRT-PCR and GFP reporter assays demonstrating that aurodox completely blocks SPI-2 expression.

Additionally, gene expression patterns of 12 additional pathogenicity islands within *S*. Typhimurium in response to aurodox were examined, revealing SPI-2 as the only downregulated pathogenicity island (electronic supplementary material, figure S3). In SPI-1 *sicA, sipB* and *hilC* were upregulated. To probe the mode of action of aurodox, we identified orthologous genes differentially expressed in response to aurodox in both EHEC [6] and *S*. Typhimurium (electronic supplementary material, figure S4). Seventeen orthologous genes were identified, with 5 upregulated and 12 downregulated. Several genes have previously shown to be involved in virulence regulation, including the alcohol dehydrogenase-encoding *adhE*. Previous data showed *adhE* null mutants poorly express the LEE in EHEC [9]. Additionally, multiple amino acid biosynthesis genes were upregulated including the *leu* operon encoding leucine biosynthesis and *met* operon encoding methionine. To establish a clear link between their altered expression and the observed modulation of virulence will require further experiments.

Of the five T3SSs examined in this study, aurodox was found to inhibit effector expression in three, all of which clustered within the SPI-2 and Ysc phylotypes. In addition, the LEE-encoded T3SSs from EPEC, EHEC and *C. rodentium*, which were found to be downregulated by aurodox in our previous study, can also be assigned to the SPI-2 phylogroup [10]. From a phylogenetic tree constructed using core T3SS proteins from multiple GNPs (figure 2*c*), we observed that the SPI-2 and Ysc T3SS cluster within one clade. This suggests that phylogeny may well be a predictor of aurodox activity. To test this, more expansive testing of species within the SPI-2/Ysc clade is required. We note that *V. parahemolyticus* TTSS2 was the exemption to this. This T3SS is unusual enough to have been excluded from previous phylogenies [10], despite individual genes being Ysc-type. Whether it is structural differences between the two systems or a result of cross-regulation between the two systems that resulted in a lack of inhibition remains to be elucidated.

Finally, it has recently been reported that PurA is the target through which aurodox exerts its antivirulence effects in both EHEC and *S*. Typhimurium. In our RNA-Seq data, the *purA* gene was not significantly differentially expressed in either EHEC or *Salmonella*.

## 4. Conclusion

We have shown that aurodox selectively inhibits expression of specific T3SS in different pathogens. In this work, we demonstrate activity against *S*. Typhimurium, *Y. pseudotuberculosis* and *V. parahaemolyticus*. Aurodox does not block all T3SS and has a specific inhibitory effect on the SPI-2 T3SS in *S*. Typhimurium. We also note phylogroup-dependent activity limited to SPI-2/Ysc phylogroups. The research highlights the potential of aurodox as a promising compound to combat pathogens by targeting virulence mechanisms.

## Data Availability

RNA-seq data are accessible on the short read archive (https://www.ebi.ac.uk/ena/browser/home) under accession number PRJEB74271. Supplementary material is available online [11].
